# Patient-derived organoid elucidates the identical clonal origin of bilateral breast cancer with diverse molecular subtypes

**DOI:** 10.3389/fonc.2024.1361603

**Published:** 2024-05-10

**Authors:** Zhongbin Han, Liangxue Yao, Yanhua Fang, Sijing Chen, Ruiqing Lian, Yongqiang Yao, Hongsheng Chen, Xuening Ji, Weiting Yu, Zhe Wang, Ruoyu Wang, Shanshan Liang

**Affiliations:** ^1^ The Key Laboratory of Biomarker High Throughput Screening And Target Translation of Breast and Gastrointestinal Tumor, Affiliated Zhongshan Hospital of Dalian University, Dalian, Liaoning, China; ^2^ Breast and Thyroid Surgery, Affiliated Zhongshan Hospital of Dalian University, Dalian, Liaoning, China; ^3^ Pathology Department, Affiliated Zhongshan Hospital of Dalian University, Dalian, Liaoning, China; ^4^ Oncology Department, Affiliated Zhongshan Hospital of Dalian University, Dalian, Liaoning, China

**Keywords:** organoids, bilateral breast cancer, driver mutations, evolution, molecular subtypes

## Abstract

Bilateral breast cancer (BBC), an infrequent breast cancer subtype, has primarily been studied in terms of incidence, prognosis, and through comparative analysis of synchronous (SBBC) and metachronous (MBBC) manifestations. The advent and application of organoid technology hold profound implications for tumor research and clinical management. This study represents the pioneering use of organoid models in BBC research. We established organoid lines from two surgical tumor specimens of a BBC patient, with one line undergoing detailed pathological and genomic analysis. The BBC organoid from the right breast demonstrated a marker expression profile of ER (-), PR (-), HER-2 (0), and Ki67 index 10%, indicating that it may derived from the TNBC tissue. Whole Exome Sequencing (WES) displayed consistent set of Top10 cancer driver genes affected by missense mutations, frameshift mutation, or splice site mutations in three tumor tissues and the organoid samples. The organoids’ single nucleotide polymorphisms (SNPs) were more closely aligned with the TNBC tissue than other tumor tissues. Evolutionary analysis suggested that different tumor regions might evolve from a common ancestral layer. In this case, the development of BBC organoids indicated that simultaneous lesions with diverse molecular profiles shared a high degree of consistency in key tumor-driving mutations. These findings suggest the feasibility of generating BBC organoids representing various molecular types, accurately replicating significant markers and driver mutations of the originating tumor. Consequently, organoids serve as a valuable *in vitro* model for exploring treatment strategies and elucidating the underlying mechanisms of BBC.

## 1 Introduction

Breast cancer has surpassed lung cancer making the most pervasive malignancy worldwide, according to the latest global statistics (1). Bilateral breast cancer (BBC), defined as cancers occurring simultaneously or sequentially in bilateral breast tissue, is an uncommon breast cancer with the incidence 2-11% of all breast cancer. According to the time interval of presentation from the first tumor, BBC is mainly divided into synchronous bilateral breast cancer (SBBC) and metachronous bilateral breast cancer (MBBC) (2, 3). The interval time varies from 1~24 months lacking uniform standard, while six months is adopted by most studies (3–6). Treatment protocols for both SBBC and MBBC align with those for unilateral breast cancer, typically involving surgery complemented by radiotherapy, chemotherapy, endocrine, and targeted therapies. In determining adjuvant treatments, clinicians often consider either the more advanced stage or the most severe histological features of the tumors (7). However, the implications of adjuvant therapy on BBC incidence remain inadequately explored. For instance, a study involving 6,550 BBC cases revealed a paradoxical dual effect of adjuvant therapy on MBBC, where it appeared to reduce risk while potentially exacerbating prognosis (4). This underscores the urgent need to understand BBC’s etiology and refine adjuvant treatment strategies.

In the realm of tumor research and clinical treatment, the emergence and utilization of organoid technology mark a significant milestone. Breast cancer organoids develop somewhat late compared to other cancers, such as colorectal cancer (8), pancreatic cancer (9), prostate cancer (10), and liver cancer (11). A breakthrough was achieved in 2018 when Hans Clevers and Sachs refined culture conditions, establishing over a hundred long-term cultured breast cancer patient-derived organoids (PDOs). These organoids have since become instrumental in cancer research, high throughput drug screening, and the evaluation of drug responses in personalized medicine (12). Subsequent studies have advanced this field further by co-culturing breast cancer organoids with other cells like fibroblasts or epithelial cells, thereby better mimicking the *in vivo* microenvironment, drug response, and invasion processes of breast cancer (13, 14). Investigations into the ultrastructure have also shed light on the metabolic and secretory activity differences between breast cancer and normal tissue-derived organoids (15), significantly enhancing our understanding of breast cancer’s development and heterogeneity. Yet, reports on the application of organoids in BBC research have been conspicuously absent, with most studies concentrating on incidence, prognosis, or comparative analyses between SBBC and MBBC (6, 7, 16). Few have delved into treatment methodologies or underlying mechanisms. Given the spontaneous and heterogeneous nature of BBC, and challenges in developing suitable animal models, breast cancer organoids currently present the most promising avenue for addressing these research gaps.

In this study, we established two organoid lines from the surgical tumor tissues of an SBBC patient who underwent modified radical mastectomy. Notably, the organoids derived from the right breast were found to retain key driver mutations and exhibited a linear evolution alongside all sampled BBC tissues (including one from the left breast and two from the right), confirmed organoids serving as a valuable *in vitro* model for elucidating the underlying mechanisms of BBC.

## 2 Materials and methods

### 2.1 Patient and sample collection

**Figure 1:**
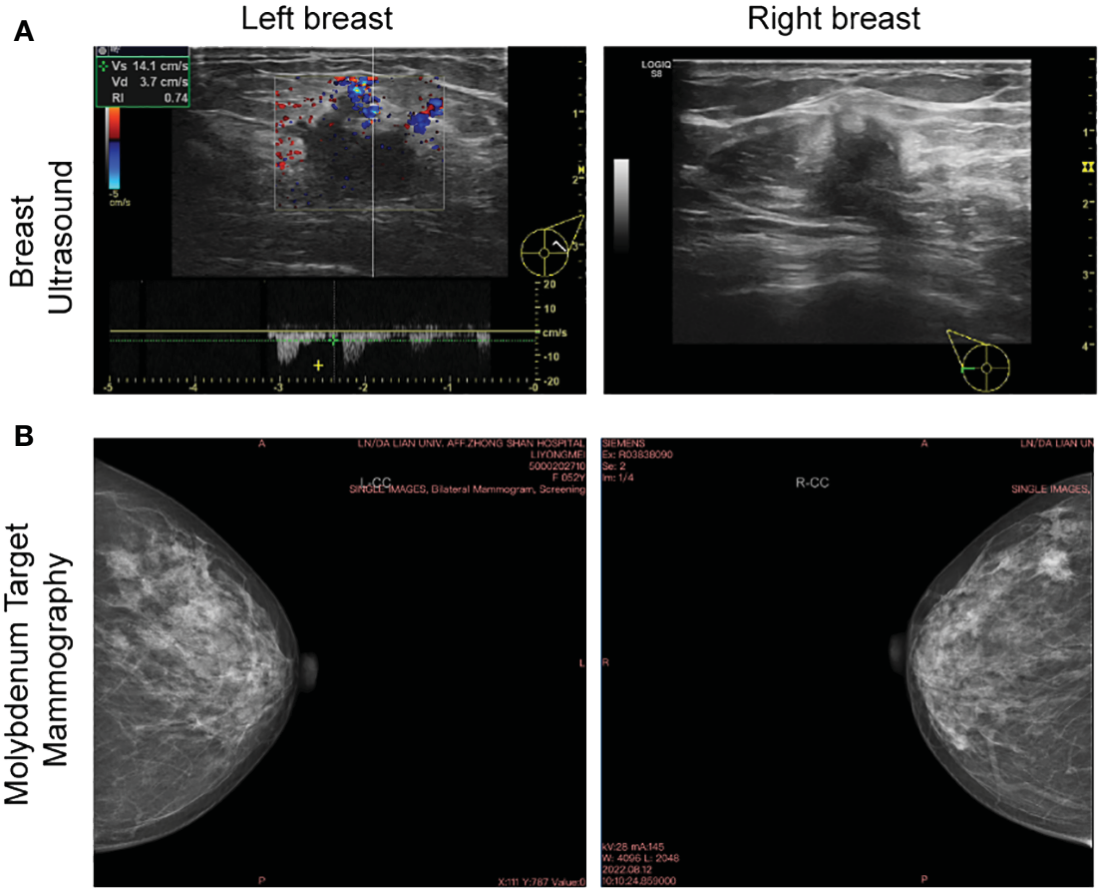
Breast ultrasound and molybdenum target of bilateral breast. **(A)** The baseline breast ultrasound performed on Aug. 12, 2022 showed solid masses in bilateral breast. **(B)** Molybdenum target performed on the same day showed nodule in right breast and bilateral mammary gland hyperplasia with benign calcification of the left breast.

**Figure 2:**
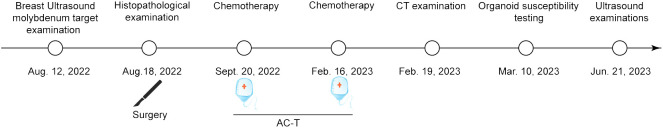
Timeline of the diagnostic and therapeutic process.

The present study has been approved by the Ethics Committee of Affiliated Zhongshan Hospital of Dalian University (Dalian, China) with Institutional Review Board approval number KY2023-075-1 and it conforms to the provisions of the Declaration of Helsinki. Written consent was obtained from the patient prior to collection of tissue and clinical data stored in a de-identifed manner. A 52-year-old Chinese woman was admitted with bilateral breast mass in Affiliated Zhongshan Hospital of Dalian University on August 15, 2022. The patient discovered lumps in both breasts 10 days ago, with no pain, no nipple discharge, nor change in the appearance. The breast ultrasound demonstrated that solid masses (BI-RADS 4c, BI-RADS 4a) and low-echo (BI-RADS 3) in the right breast, solid masses (BI-RADS 4a, BI-RADS 4c) and multiple low-echo (BI-RADS 4a) the left. No axillary lymph nodes (LN) were palpable (**Figure 1A**). Molybdenum target mammography showed nodule in right breast (BI-RADS 4c) and bilateral mammary gland hyperplasia with benign calcification of the left breast (**Figure 1B**). Surgery of bilateral breast modified radical mastectomy and sentinel lymph node biopsy was conducted on August 18, 2022. Postoperative pathology results reported there were three carcinoma lesions: invasive breast cancer (right breast, moderately differentiation), Low-grade fibromatosis-like spindle cell carcinomas (right breast, well differentiated), invasive breast cancer (left breast, moderately differentiation) and no axillary lymph node metastasis (0/19) existed. This patient completed the 7-week AC-T chemotherapy regimen on February 19, 2023 and no metastatic lesions were found throughout the body. The latest ultrasound on various lymph nodes was performed on June 21, 2023. The timeline of the diagnostic and therapeutic process was shown in **Figure 2**.

Two tumor samples (one taken from the right breast, the other from the left) were obtained from this patient during surgery and kept in AdDF++++ [Advanced DMEM/F12 (Gibco, New York, USA) containing 1× Glutamax (Gibco, New York, USA), 10 mM HEPES (Sigma-Aldrich, Darmstadt, Germany), 5µM Y-27632 (Selleck, Texas, USA) and P/S (Sigma-Aldrich, Darmstadt, Germany)] transported directly to the laboratory. The research protocol was approved and recorded by the Ethics Committee of Affiliated Zhongshan Hospital of Dalian University (KY2023-075-1). All procedures are carried out in accordance with the Helsinki Declaration.

### 2.2 Organoid culture

The surgical samples were cut into small pieces, and followed by digested in AdDF++++ with collagenase IV (5 mg/mL, Gibco, New York, USA), DNase I (100 µg/mL, Sigma-Aldrich, Darmstadt, Germany) solution for 3~4 hour at 37°C in water bath. When large tissue disappears, the cell suspension was strained over a 70 μm filter before centrifugation at 1400 rpm for 5 min. The cell pellet was washed three times with D-PBS and if red precipitate was observed, the pellet was lysed in 1 mL of red blood cell lysis buffer (Invitrogen, Carlsbad, CA, USA) for 5 min and centrifuged at 1400 rpm. Then cell suspension was mixed with Matrigel (Corning, New York, USA) by the ratio 1:1 (vol/vol). 50 μL droplets of Matrigel-cell suspension droplets were added to a preheated 24-well. After gelation, 500 μL breast cancer organoid medium (detailed composition seen **Table 1**) was gently added to each well. The medium was changed every 3 days. Passaging process was conducted 1-2 weeks. 1 mL/per well of TrypLE Express (Gibco, New York, USA) was added, incubated for 10 min at 37°C. Then, 1.5 mL AdDF++++ was introduced to stop digestion and centrifugated at 1400 rpm. The pellets were suspended in cold Matrigel and reseeded in the ratio 1:2~1:4 as described above.

**Table 1:** Composition of culture medium for breast cancer organoid.

Reagents	Supplier	Catalogue number	Final Concentration
R-Spondin-3	Peprotech	120-44	250 ng/mL
Noggin	Peprotech	120-10C	100 ng/mL
FGF10	Peprotech	100-26	20 ng/mL
FGF7	Peprotech	100-19	5 ng/mL
EGF	Peprotech	AF-100-15	5 ng/mL
Heregulin β1	Peprotech	100-03	5 nM
A83-01	Tocris	2939	500 nM
SB202190	Sellerk	S1077	500 nM
B27 supplement	Gibco	17504-44	1×
Nicotinamide	Sigma	N0636	10 mM
N-Acetylcysteine	Sigma	A9165	1.25 mM
Primocin	Ant-pm-1	Invivogen	50 μg/mL
AdDF++++	–	–	–

### 2.3 Histology and imaging

After fixed with 4% paraformaldehyde, Matrigel-organoids was aspirated from the 24-well plates into a 1.5 mL EP tube and centrifuged at 1000 rpm for 5 min, then embedded in 2% agarose. The solidified organoids-agarose was fixed in 4% paraformaldehyde overnight, and followed by gradient dehydration the next day, treated with dimethylbenzene for 40 min before embedded in paraffin. 4 μm paraffin sections were cleared with dimethylbenzene and rehydrated with gradient ethanol. For Immunohistochemistry (IHC) assay, the paraffin section antigen retrieval was performed by microwave heating and stained with the following antibodies: Rabbit anti-ER antibody (Santa, Dallas, Texas, USA, sc-8002, 1:300), anti-PR (CST, Danvers, MA, USA, 8757s, 1:600), anti-HER2 (CST, 2165s, 1:600), and anti-Ki67 (CST, 9449s, 1:800). DAB kit was purchased from Zhongshan Goldenbridge Biotechnology Company (Beijing, China). Nuclei were counterstained with haematoxylin. Images were acquired with Leica Eclipse E600 microscope.

### 2.4 DNA extraction and whole-exome sequencing analysis

One organoid line from the right breast and three formalin-fixed & paraffin-embedded (FFPE) primary tumor sample were subjected to whole-exome sequencing by sequencing company (BGI, ShenZhen, China). Whole-Exome Sequencing (WES) libraries were sequenced with paired-end (2 × 100 bp) runs using BGISEQ-500 to depths of ~250 × (about 33 GB per sample). Human reference genome GRCh38 was aligned by WES data, with the Burrows–Wheeler Aligner v0.7.1 using the default options. The data was following processed by the Genome Analysis Toolkit (GATK). After obtaining a highly reliable mutation set, Annodb was used to annotate the mutations. Copy number variations (CNVs) and visualization were implemented by CNVkit. Then we compared mutations with known driver genes in databases (17, 18) and literatures (19–22) to screen the known tumor driver genes. Proportions of SNPs and types of base substitutions were counted. Variations of tumor tissues and matched organoids were plotted using Venn plots. The data processed by GATK was also analyzed by SuperFreq to generate riverplots for tumor evolution, taking organoid genetic information as the germline. SuperFreq is an R software package for the analysis of cancer exomes. It analyzes and filters somatic SNVs and short indels, copy numbers, and tracks clones from multiple samples. SuperFreq does not require a matched normal sample and relies on uncorrelated controls. When analyzing multiple samples from a patient, SuperFreq cross-checks variant calling to improve clone tracking, which helps distinguish between somatic and germline variants.

## 3 Results

### 3.1 Establishing patient−derived organoids

**Figure 3:**
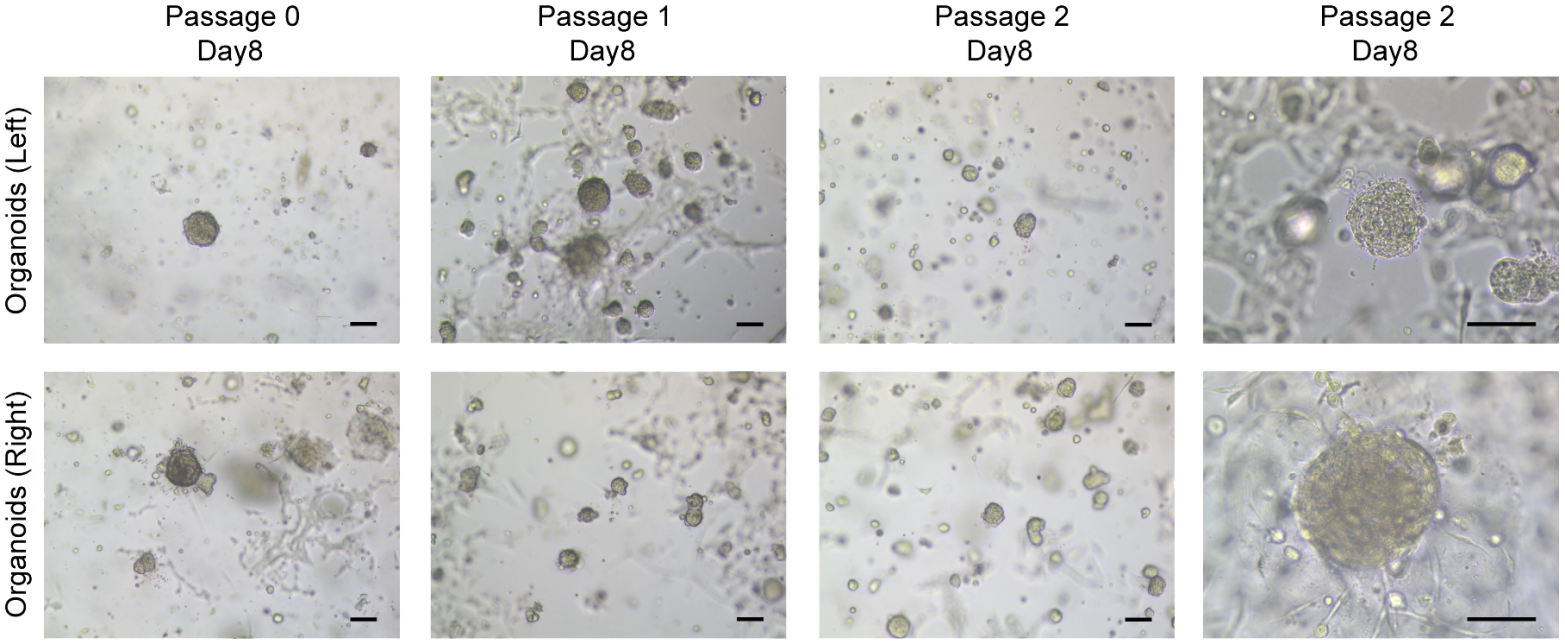
Breast cancer organoids established from a SBBC patient. Representative images of breast cancer organoids from bilateral breast. Scale bar = 100 μm.

Two PDO lines were established from surgery tissues. Tumor tissue from the left breast was very small, only 1.1 × 10^5^ cells were obtained after digestion, while the number of the right was 4.4 × 10^5^. Subsequent inoculation led to the monitoring of each organoid generation under bright-field microscopy. Morphologically, there were no substantial differences noted between the organoids derived from the bilateral breast tissues. Predominantly, they exhibited a compact, spheroid form, with a subset of cells adhering to the Matrigel dome’s base and extending outward in a star-like pattern (**Figure 3**). By the 14th day of the second passage, both organoid lines were harvested, either embedded in wax blocks or cryopreserved for sequencing purposes. Notably, despite the robust growth of the organoids originating from the left breast tumor, insufficient quantities were harvested for comprehensive pathological analysis and genetic profiling, likely attributed to the limited cell count at the outset.

### 3.2 BBC organoids match the original histological characteristics

**Figure 4:**
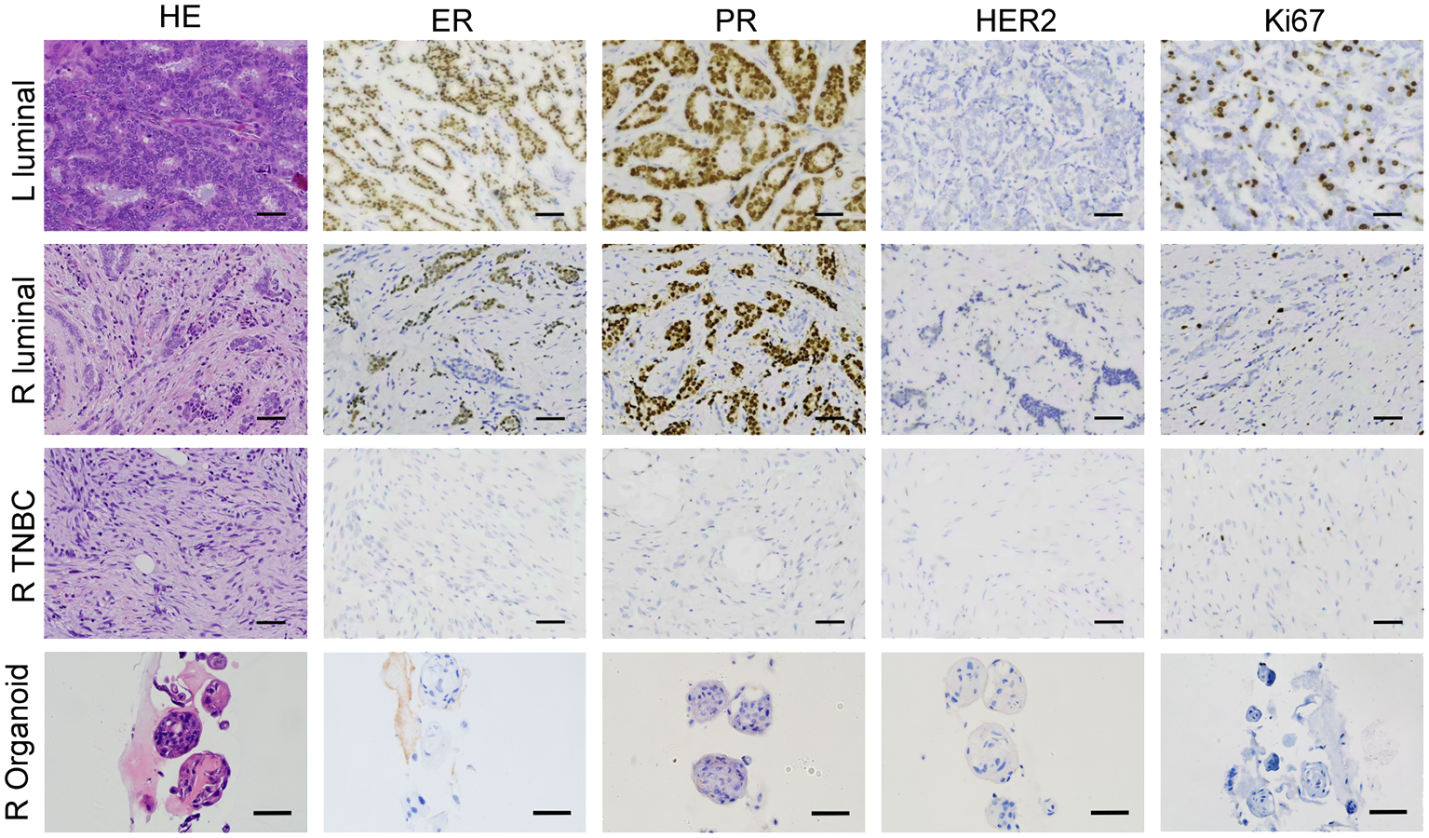
BBC organoids match the original histological characteristics. Representative images of H&E and immunohistochemistry staining of three breast tumor tissues together with the organoids derived from the TNBC tissue from the right breast. Scale bar = 50 μm.

The organoid cultures were established using two tumor tissue samples, one each from the patient’s left and right breasts. These samples, along with additional tissues from the right breast, underwent clinical pathological diagnosis. The postoperative pathology results revealed the following: 1. Invasive breast cancer in the left breast, exhibiting moderate differentiation, with ER (80%+), PR (90%+), HER-2 (0), and a Ki67 index of 30%. 2. Invasive breast cancer in the right breast, also showing moderate differentiation, with ER (80%+), PR (90%+), HER-2 (0), and a Ki67 index of 10%. 3. A low-grade, well-differentiated fibromatosis-like spindle cell carcinoma in the right breast, characterized by ER (-), PR (-), HER-2 (0), and a Ki67 index of 10% (as illustrated in **Figure 4**). The organoid that was successfully established from the right breast demonstrated a marker expression profile of ER (-), PR (-), HER-2 (0), and Ki67 index 10%. These findings were in alignment with the molecular pathological traits of triple negative breast cancer (TNBC) in the right breast.

### 3.3 Organoids from one tumor tissue retain key driver mutations and evolved linearly with all the BBC tissues

**Figure 5:**
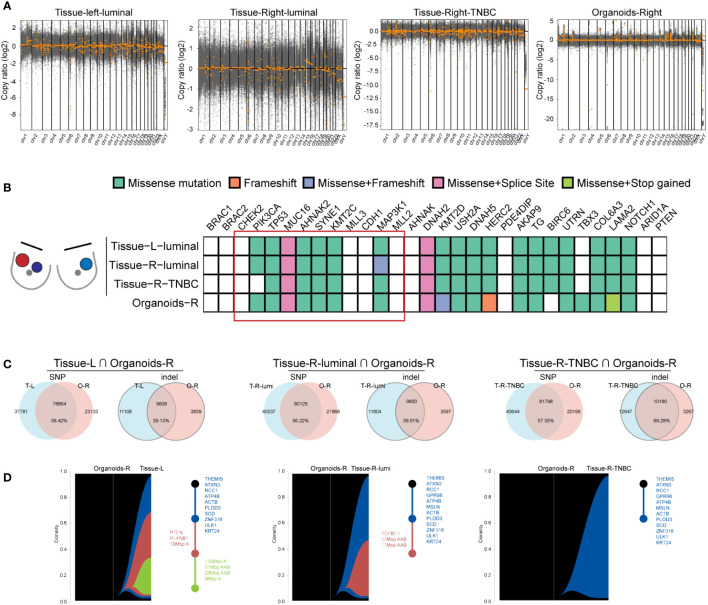
Organoids from one tumor tissue retain key driver mutations and evolved linearly of all the BBC tissues. **(A)** Scatterplots illustrating genome CNAs of tissues and organoids. DNA copy number gains (above 0) and losses (below 0) found in the three BC tissues are different and the CNAs of organoids are closer to TNBC sample. **(B)** Heat-map analysis of the top 30 somatic mutations affecting cancer genes in three BBC tissues and organoids derived from one of the breast cancer tissues. **(C)** Venn diagrams indicating the number of SNP and indel present in each BBC tissue and the organoids-R. **(D)** Riverplots generated by SuperFreq analysis, denoting clonal evolution of organoids cultured from organoids-R and three BBC tissues (left).

To ascertain if the organoid line from the right breast retained the genetic mutations present in the original tumor, and to evaluate its genetic similarity to the other two tumor tissues, Whole Exome Sequencing (WES) was performed on the organoids and three BBC tissues. Notably, the copy number variation (CNV) patterns of the three breast cancer tissues differed significantly, with the organoids exhibiting a more constrained CNV range compared to the tissues. The copy number variation (CNV) of three BC tissues varied significantly from each other and the organoids’ CNV is smaller than tissues. According to the variety range (-20~5), CNV of organoids was closer to the right breast TNBC (-17.5~0) (**Figure 5A**), which was consistent with pathological results in **Figure 4**. On the basis of mutated genes in cBioPortal database, which was targeted sequencing of 2509 primary breast tumors with 548 matched normal tissues (23, 24), a total of 30 genes including BBC driver mutations BRCA1, BRCA2, CHEK2 and other high frequency mutations in breast cancer were represented. The heatmap displayed consistent set of Top10 cancer driver genes (highlighted in a red box) affected by missense mutations, frameshift mutation, or splice site mutations in three tumor tissues and one organoid samples. Meanwhile, diversity mutations among the samples also existed (**Figure 5B**). Further, we delved into the similarity of SNP and indel alterations between the organoids and tissue samples. The shared SNP alterations between the organoids and left BC tissue, right luminal BC tissue, and right TNBC tissue were 78,864 (56.42%), 80,129 (56.22%), and 81,798 (57.35%), respectively. The shared indel alterations were 9,608 (39.13%), 9,850 (39.01%), and 10,180 (69.28%), respectively, suggesting a significant match between the organoids and the TNBC tissue, as shown in **Figure 5C**. Additionally, in order to see if the lesion tissues at three different locations of the simultaneous BBC had the same origin in clonal evolution, and whether the organoids we cultured could capture this phenomenon. tumor evolution was analyzed by examining the organoid culture alongside the TNBC and the three BBC tissues, using a recent algorithm designed to track clonal evolution. Riverplots generated by SuperFreq analysis, denoting clonal evolution of organoids cultured from organoids-R and three BBC tissues (left). The y axis denotes the proportion of tumor cells in each subclone. The somatic mutations detected in all tumor cells across the organoids-R and tissue are denoted in dark blue. Subclones present in tissues in varying proportions are denoted in different colors. Visualization of the riverplots as reconstructed phylogenetic trees (Right). Each node represents a clonal population. Representative cancer driver genes are shown by the node they belong to and according to the colors representing that clonal population in the riverplots. This analysis revealed that organoids established from a single BBC tissue evolved along the same trajectory, sharing common early driver mutations, including THEMIS, ATXN3, RCC1, ATP4B, PLOD3, SCD, ZNF318, ULK1, KRT24, with all cancer tissues, as illustrated in **Figure 5D**. Collectively, these findings suggest that organoids derived from a singular tumor tissue preserve key driver mutations and exhibit a linear evolution consistent with all sampled BBC tissues.

## 4 Discussion

Bilateral Breast Cancer (BBC) encompasses two subtypes: Bilateral Primary Breast Cancer (BPBC) and Bilateral Metastatic Breast Cancer (MBBC). A targeted sequencing analysis of 254 genes in 49 BBC patients revealed that only 6% of BBC cases were metastatic (25). Consequently, in women with a history of breast cancer, a contralateral breast cancer is conventionally regarded as an independent BPBC (26). In this study, the patient’s cancer was classified as BPBC based on sequencing outcomes and pathological assessments. The incidence of BBC is on the rise, attributed to advancements in imaging techniques and improved survival rates in unilateral breast cancer patients. Some risk factors, such as lobular histology, young age at diagnosis, family history, driver gene mutations (BRCA1, BRCA2, CHEK2) are considered as high triggers (27, 28). According to cBioPortal database, the mutation frequency of BRCA1, BRCA2, CHEK2 are 1.7%, 1.8%, 0.7%, respectively (23, 24). While, no mutation of these three genes was found in tissue samples and organoids in this patient, probably due to the low mutation frequency of these genes.

BBC has different histological types and nuclear differentiation, so that different lesions may have inconsistent responses to the same treatment regimen. The existing adjuvant treatment are based on higher stage or the most adverse pathological characteristics without considering the response of other lesions (7), which may be the main reason for the failure of dual breast cancer treatment. Taking this as a consideration, organoids will be an excellent model for studying BBC, because of BBCs’ affected by the same germline genetics and environmental exposures (29), thus organoid derived from one tissue should contain most of the tumor subgroups of BBC theoretically. In fact, from our WES results, the somatic mutations of organoids are more abundant than any other BBC samples **Figure 5B**. However, if available, a better choice is to establish organoids with different lesions. The organoid model has great advantages in the study of BBC. The clinical relevance of breast cancer 2D cell lines to individual patients is not high (30). The PDX model needs to establish BBC cancer foci in different mice, however, there are individual differences between different mice. Organoid models can provide the same extracellular matrix and growth factors for different tissues from BBC, so that could overcome the interference caused by the external environment. Moreover, PDX-derived products are inefficient and time-consuming, which makes them unable to contribute quickly to individualized treatment of patients on a large scale. Therefore, it is necessary to develop organoid models of BBC to understand the biological function of somatic mutations and their role in disease progression, and to provide guidance for the clinical formulation of rational treatment strategies.

A limitation of our study is the reliance on multiple organoid cultures from the same time frame, necessitating the use of SBBC samples, which are rare. Consequently, we have so far only obtained consent from one eligible patient to provide tumor samples. Additionally, the prioritization of pathological diagnosis limited our ability to culture organoids from more than two tumor samples. Operational constraints meant that only one sample successfully underwent the complete organoid experimentation, including establishment and characterization, due to insufficient initial cell quantities for wax embedding and sequencing. Future research will focus more on SBBC patient samples to amass more comprehensive data. Furthermore, a noteworthy issue in the cultivation and research of breast cancer organoids deserves attention. The propensity for hormonal receptor loss during culture within current systems. According to current reports, this may be due to the inability of existing breast cancer organoid culture systems to accommodate the highly heterogeneous patient tissue sources, leading to the loss of ER and PR in some tissues or after passage intervals (12, 31). In the context of this research, the organoid utilized for IHC analysis originates from the P1 generation, and it was successfully cultivated from the right breast, demonstrating a marker expression profile indicative of ER (-), PR (-), HER-2 (0), and a Ki67 index of 10%, mirroring the molecular pathological hallmarks of TNBC. Nonetheless, the potential modulatory effects of ex vivo organoid culture on the originating tissues remain a consideration. Addressing this challenge, there is a burgeoning interest among scholars in identifying and exploring remedial strategies (32). For future applications, we can simultaneously test the effectiveness of multiple drugs in organoids cultured from different lesions of BBC patients, and select a better treatment regimen for multiple organoids to provide guidance and suggestions for clinical practice.

## 5 Conclusion

In summary, our study successfully established organoids from two SBBC tissue samples, integrating clinical, pathological, and genomic data via WES technologies. Organoids derived from a singular tumor tissue preserve key driver mutations and exhibit a linear evolution consistent with all sampled BBC tissues. This approach preliminarily demonstrates the utility of organoid technology in BBC research. Overall, our findings suggest that BBC organoids could serve as a valuable tool in understanding the development and evolution of BBC.

## Data availability statement

The datasets presented in this study can be found in online repositories. WES data generated for this study have been deposited in the Genome Sequence Achieve at the National Genomics Data Center. This data can be found here: https://bigd.big.ac.cn/gsa-human/browse/HRA006501. The names of the repository/repositories and accession number(s) can be found in the article/supplementary material.

## Ethics statement

The studies involving humans were approved by The Ethics Committee of Affiliated Zhongshan Hospital of Dalian University. The studies were conducted in accordance with the local legislation and institutional requirements. The participants provided their written informed consent to participate in this study. Written informed consent was obtained from the individual(s) for the publication of any potentially identifiable images or data included in this article.

## Author contributions

ZH: Writing – review & editing, Resources, Investigation. LY: Data curation, Investigation, Methodology, Writing – review & editing. YF: Writing – review & editing, Writing – original draft, Investigation, Data curation. SC: Writing – review & editing, Methodology, Data curation. RL: Writing – review & editing, Methodology, Data curation. YY: Writing – review & editing, Resources, Investigation. HC: Writing – review & editing, Investigation. XJ: Writing – review & editing, Investigation. WY: Investigation, Writing – review & editing. ZW: Writing – review & editing, Investigation. RW: Writing – review & editing, Supervision, Funding acquisition. SL: Writing – review & editing, Supervision, Project administration, Conceptualization.

## References

[1] Sung H Ferlay J Siegel R Laversanne M Soerjomataram I Jemal A. Global cancer statistics 2020: GLOBOCAN estimates of incidence and mortality worldwide for 36 cancers in 185 countries. CA: Cancer J Clin. (2021) 71:209–49. doi: 10.3322/caac.21660 33538338

[2] Wadasadawala T Lewis S Parmar V Budrukkar A Gupta S Nair N. Bilateral breast cancer after multimodality treatment: A report of clinical outcomes in an Asian population. Clin Breast Cancer. (2018) 18:e727–37. doi: 10.1016/j.clbc.2017.11.003 29254601

[3] Pan B Xu Y Zhou YD Yao R Wu HW Zhu QL. The prognostic comparison among unilateral, bilateral, synchronous bilateral, and metachronous bilateral breast cancer: A meta-analysis of studies from recent decade, (2008-2018). Cancer Med. (2019) 8:2908–18. doi: 10.1002/cam4.2198 PMC6558468 31038845

[4] Hartman M Czene K Reilly M Adolfsson J Bergh J Adami HO. Incidence and prognosis of synchronous and metachronous bilateral breast cancer. J Clin Oncol. (2007) 25:4210–6. doi: 10.1200/JCO.2006.10.5056 17878475

[5] Jobsen J van der Palen J Ong F Riemersma S Struikmans H. Bilateral breast cancer, synchronous and metachronous; differences and outcome. Breast Cancer Res Treat. (2015) 153:277–83. doi: 10.1007/s10549-015-3538-5 26268697

[6] Ozturk A Alco G Sarsenov D Ilgun S Ordu C Koksal U. Synchronous and metachronous bilateral breast cancer: A long-term experience. J B.U.ON. (2018) 23:1591–600. 30610782

[7] Holm M Tjonneland A Balslev E Kroman N. Prognosis of synchronous bilateral breast cancer: a review and meta-analysis of observational studies. Breast Cancer Res Treat. (2014) 146:461–75. doi: 10.1007/s10549-014-3045-0 25007962

[8] Sato T Stange D Ferrante M Vries R Van Es J Van den Brink S. Long-term expansion of epithelial organoids from human colon, adenoma, adenocarcinoma, and Barrett's epithelium. Gastroenterology. (2011) 141:1762–72. doi: 10.1053/j.gastro.2011.07.050 21889923

[9] Boj S Hwang C Baker L Chio I Engle D Corbo V. Organoid models of human and mouse ductal pancreatic cancer. Cell. (2015) 160:324–38. doi: 10.1016/j.cell.2014.12.021 PMC4334572 25557080

[10] Park J Lee J Phillips J Huang P Cheng D Huang J. Prostate epithelial cell of origin determines cancer differentiation state in an organoid transformation assay. Proc Natl Acad Sci U S A. (2016) 113:4482–7. doi: 10.1073/pnas.1603645113 PMC4843433 27044116

[11] Broutier L Mastrogiovanni G Verstegen M Francies H Gavarró L Bradshaw C. Human primary liver cancer-derived organoid cultures for disease modeling and drug screening. Nat Med. (2017) 23:1424–35. doi: 10.1038/nm.4438 PMC5722201 29131160

[12] Sachs N de Ligt J Kopper O Gogola E Bounova G Weeber F. A living biobank of breast cancer organoids captures disease heterogeneity. Cell. (2018) 172:373–86.e310. doi: 10.1016/j.cell.2017.11.010 29224780

[13] Chatterjee S Bhat V Berdnikov A Liu J Zhang G Buchel E. Paracrine crosstalk between fibroblasts and ER breast cancer cells creates an IL1β-enriched niche that promotes tumor growth. iScience. (2019) 19:388–401. doi: 10.1016/j.isci.2019.07.034 31419632 PMC6706609

[14] Parigoris E Lee S Mertz D Turner M Liu A Sentosa J. Cancer cell invasion of mammary organoids with basal-in phenotype. Advanced healthcare materials. (2021) 10:e2000810. doi: 10.1002/adhm.202000810 32583612 PMC7759600

[15] Signati L Allevi R Piccotti F Albasini S Villani L Sevieri M. Ultrastructural analysis of breast cancer patient-derived organoids. Cancer Cell Int. (2021) 21:423. doi: 10.1186/s12935-021-02135-z 34376194 PMC8353820

[16] Sakai T Ozkurt E DeSantis S Wong S Rosenbaum L Zheng H. National trends of synchronous bilateral breast cancer incidence in the United States. Breast Cancer Res Treat. (2019) 178:161–7. doi: 10.1007/s10549-019-05363-0 31325072

[17] Sondka Z Bamford S Cole C Ward S Dunham I Forbes S. The COSMIC Cancer Gene Census: describing genetic dysfunction across all human cancers. Nat Rev Cancer. (2018) 18:696–705. doi: 10.1038/s41568-018-0060-1 30293088 PMC6450507

[18] Martínez-Jiménez F Muiños F Sentís I Deu-Pons J Reyes-Salazar I Arnedo-Pac C. A compendium of mutational cancer driver genes. Nat Rev Cancer. (2020) 20:555–72. doi: 10.1038/s41568-020-0290-x 32778778

[19] Kandoth C McLellan M Vandin F Ye K Niu B Lu C. Mutational landscape and significance across 12 major cancer types. Nature. (2013) 502:333–9. doi: 10.1038/nature12634 PMC3927368 24132290

[20] Tamborero D Gonzalez-Perez A Perez-Llamas C Deu-Pons J Kandoth C Reimand J. Comprehensive identification of mutational cancer driver genes across 12 tumor types. Sci Rep. (2013) 3:2650. doi: 10.1038/srep02650 24084849 PMC3788361

[21] Vogelstein B Papadopoulos N Velculescu V Zhou S Diaz L Kinzler K. Cancer genome landscapes. Sci (New York N.Y.). (2013) 339:1546–58. doi: 10.1126/science.1235122 PMC3749880 23539594

[22] Bailey M Tokheim C Porta-Pardo E Sengupta S Bertrand D Weerasinghe A. Comprehensive characterization of cancer driver genes and mutations. Cell. (2018) 174:1034–5. doi: 10.1016/j.cell.2018.07.034 PMC8045146 30096302

[23] Curtis C Shah SP Chin SF Turashvili G Rueda OM Dunning MJ. The genomic and transcriptomic architecture of 2,000 breast tumours reveals novel subgroups. Nature. (2012) 486:346–52. doi: 10.1038/nature10983 PMC3440846 22522925

[24] Pereira B Chin SF Rueda OM Vollan HK Provenzano E Bardwell HA. The somatic mutation profiles of 2,433 breast cancers refines their genomic and transcriptomic landscapes. Nat Commun. (2016) 7:11479. doi: 10.1038/ncomms11479 27161491 PMC4866047

[25] Begg CB Ostrovnaya I Geyer FC Papanastasiou AD Ng CKY Sakr RA. Contralateral breast cancers: Independent cancers or metastases? Int J Cancer. (2018) 142:347–56. doi: 10.1002/ijc.31051 PMC5749409 28921573

[26] Alkner S Tang M Brueffer C Dahlgren M Chen Y Olsson E. Contralateral breast cancer can represent a metastatic spread of the first primary tumor: determination of clonal relationship between contralateral breast cancers using next-generation whole genome sequencing. Breast Cancer research: BCR. (2015) 17:102. doi: 10.1186/s13058-015-0608-x 26242876 PMC4531539

[27] Narod SA. Bilateral breast cancers. Nat Rev Clin Oncol. (2014) 11:157–66. doi: 10.1038/nrclinonc.2014.3 24492834

[28] O'Brien JA Ho A Wright GP Stempel M Patil S Krause K. Breast-conserving surgery in bilateral breast cancer. Ann Surg Oncol. (2015) 22:3389–96. doi: 10.1245/s10434-015-4746-2 PMC4636081 26265365

[29] Hamy AS Abecassis J Driouch K Darrigues L Vandenbogaert M Laurent C. Evolution of synchronous female bilateral breast cancers and response to treatment. Nat Med. (2023) 29:646–55. doi: 10.1038/s41591-023-02216-8 PMC10033420 36879128

[30] Sharma SV Haber DA Settleman J. Cell line-based platforms to evaluate the therapeutic efficacy of candidate anticancer agents. Nat Rev Cancer. (2010) 10:241–53. doi: 10.1038/nrc2820 20300105

[31] D. JF v.V. EsméeJ Norman S R. JM Oded K R. HG. Long-term culture, genetic manipulation and xenotransplantation of human normal and breast cancer organoids. Nat Protoc. (2021) 16:1936–1965. doi: 10.1038/s41596-020-00474-1 PMC8221035 33692550

[32] O. MUJ Dipikaa A M. SK. Establishing conditions for the generation and maintenance of estrogen receptor-positive organoid models of breast cancer. Breast Cancer Res. (2024) 26. doi: 10.1186/s13058-024-01798-6 PMC10979603 38553763

